# Association of continuous glucose monitoring metrics with early diabetic kidney disease in early-onset type 2 diabetes

**DOI:** 10.3389/fendo.2026.1884596

**Published:** 2026-07-22

**Authors:** Yuanshuang Jiang, Jing Kang, Yan Chen

**Affiliations:** Department of Endocrinology, Second Hospital of Jilin University, Changchun, China

**Keywords:** diabetes mellitus, type 2, early-onset, continuous glucose monitoring, diabetic nephropathies, mediation analysis

## Abstract

**Background:**

The rising prevalence of early-onset type 2 diabetes (T2DM) has become a major public health concern, with these patients facing a particularly high risk of early diabetic kidney disease (DKD). Although continuous glucose monitoring (CGM)-derived metrics have shown promise in predicting complications in later-onset T2DM, their utility in early-onset T2DM, a more aggressive phenotype characterized by rapid β-cell decline and heightened complication risk, remains unclear. Moreover, the relationship between glycemic variability and renal injury, as well as the mechanisms underlying the associations between CGM metrics and DKD, have not been fully elucidated. This study aimed to evaluate the associations of time in range (TIR), coefficient of variation (CV), and glycemic risk index (GRI) with early DKD in Chinese patients with early-onset T2DM, and to explore the potential mediating role of triglycerides.

**Methods:**

This cross-sectional study included 810 individuals with early-onset T2DM, defined as diagnosis before age 40. All participants underwent ≥7 days of CGM. Early DKD was defined as a urinary albumin-to-creatinine ratio (UACR) ≥30 mg/g with an estimated glomerular filtration rate (eGFR) ≥60 mL/min/1.73m². Multivariable logistic regression, restricted cubic splines, and mediation analysis were used to evaluate independent associations, nonlinear relationships, and the mediating role of triglycerides (TG), respectively.

**Results:**

Among the participants, 183 (22.59%) had early DKD. After full adjustment including HbA1c, higher TIR was associated with lower odds of early DKD (per 1% increase: OR 0.99, 95% CI 0.98–0.99, *P* < 0.001), whereas higher GRI was associated with increased odds (per 1-unit increase: OR 1.01, 95% CI 1.01–1.02, *P* < 0.001). A U-shaped relationship was observed between CV and early DKD risk, with the nadir observed at approximately 20–30% CV. In mediation analysis, TG statistically accounted for 13.01% of the association between TIR and early DKD, and 11.92% of the association between GRI and early DKD.

**Conclusions:**

In Chinese patients with early-onset T2DM, CGM-derived metrics are independently associated with early DKD, with TIR and GRI showing opposite associations and CV exhibiting a U-shaped relationship. The observed association involving triglycerides suggests that lipid metabolism may be a correlate of glycemic control in relation to renal injury. These findings support a multidimensional strategy integrating glucose control, glycemic stability, and lipid management for renal protection in this high-risk population.

## 1 Introduction

The global rise in obesity has been paralleled by a substantial increase in the incidence and prevalence of early-onset type 2 diabetes mellitus (T2DM) (1). In China, this presents a particularly severe dual challenge: a high baseline adult diabetes prevalence of 12.8% is compounded by a rapidly accelerating shift toward younger age at onset (2). Data indicate the annual percentage change in incidence for early-onset T2DM (ages 15-39) in the Western Pacific Region—where China is the most populous country—increased sharply from 1.71% (1999-2017) to 5.01% (2017-2021) (3). Early-onset T2DM, defined by diagnosis before age 40, represents an aggressive phenotype characterized by rapid β-cell function decline and a heightened risk of microvascular complications (4, 5). Among these, diabetic kidney disease (DKD) is the most prevalent and aggressive microvascular complication in young individuals with T2DM (5). The burden of DKD is immense, with its age-standardized prevalence in China reaching 1,053.92 per 100,000 population and projected to continue rising (6). As a leading cause of chronic and end-stage renal disease, DKD imposes a severe burden on healthcare systems and patient quality of life (7). Consequently, identifying modifiable risk factors for early-stage DKD in this expanding Chinese population with early-onset T2DM is a critical clinical priority.

While glycated hemoglobin (HbA1c) has long been regarded as the gold standard for assessing long-term glycemic control and predicting the risk of distant complications in diabetes, it possesses inherent limitations. Specifically, it fails to capture daily glycemic fluctuations, hypoglycemic episodes, and postprandial glucose excursions (8). Continuous glucose monitoring (CGM) addresses these shortcomings by enabling a reduction in both hypoglycemic and hyperglycemic events and contributing to improved patient quality of life (9). Several key metrics have been derived from CGM, including time in range (TIR), coefficient of variation (CV), and glycemic risk index (GRI). Robust evidence links these CGM metrics to diabetic complications. Specifically, higher TIR is associated with a lower risk of microvascular complications in both type 1 diabetes and later-onset type 2 diabetes, and a TIR >70% is therefore recommended as a clinical target (10, 11). In contrast, the CV, a core parameter for assessing glycemic variability, reflects glucose stability, with higher values indicating greater instability. A CV below 36% is generally recommended for individuals with diabetes (12) Elevated glycemic variability is an independent risk factor for oxidative stress and endothelial dysfunction, potentially promoting vascular injury (13). However, the relationship between CV and diabetic complications remains debated, as some studies have not found a significant association with retinopathy (14). Notably, most existing evidence is derived from populations with later-onset T2DM or type 1 diabetes. Consequently, the clinical significance and behavior of CV and composite metrics such as the GRI remain largely unexplored in the distinct population of Chinese patients with early-onset T2DM.

Recent studies have begun to delineate the relationships between CGM metrics and diabetic kidney injury in broader T2DM cohorts. A systematic review of 13,987 individuals confirmed that higher TIR is associated with a reduced risk of microvascular complications, including nephropathy (15). Furthermore, cross-sectional investigations have demonstrated significant correlations between CGM-derived TIR, GRI, and urinary markers of both glomerular (albuminuria) and tubular injury in patients with T2DM (16). Another study indicated that TIR calculated from self-monitored blood glucose aligns with CGM-derived TIR and shows a similar association with microalbuminuria risk (17).

Despite these advances, critical knowledge gaps persist, specifically concerning the high-risk population with early-onset T2DM—a group underscored by its escalating incidence (16). First, the applicability and utility value of comprehensive CGM metrics, especially the debated CV and the composite GRI, for early-stage DKD within this aggressive phenotype remain largely unexplored. Second, the nature of the relationship between CV and DKD risk is unclear; a non-linear (e.g., U-shaped) association is plausible yet uninvestigated, with significant implications for personalized management. Third, the pathophysiological pathways linking CGM metrics to renal injury, such as the potential mediating role of dyslipidemia (e.g., triglycerides), are poorly understood.

To address these gaps, this study aimed to systematically elucidate the independent associations, dose-response relationships, and mediating role of triglycerides (TG) between core CGM metrics and early-stage DKD in patients with early-onset T2DM, in order to provide scientific evidence for early risk stratification and precise intervention within this high-risk population in China.

## 2 Materials and methods

### 2.1 Study population

**Figure 1:**
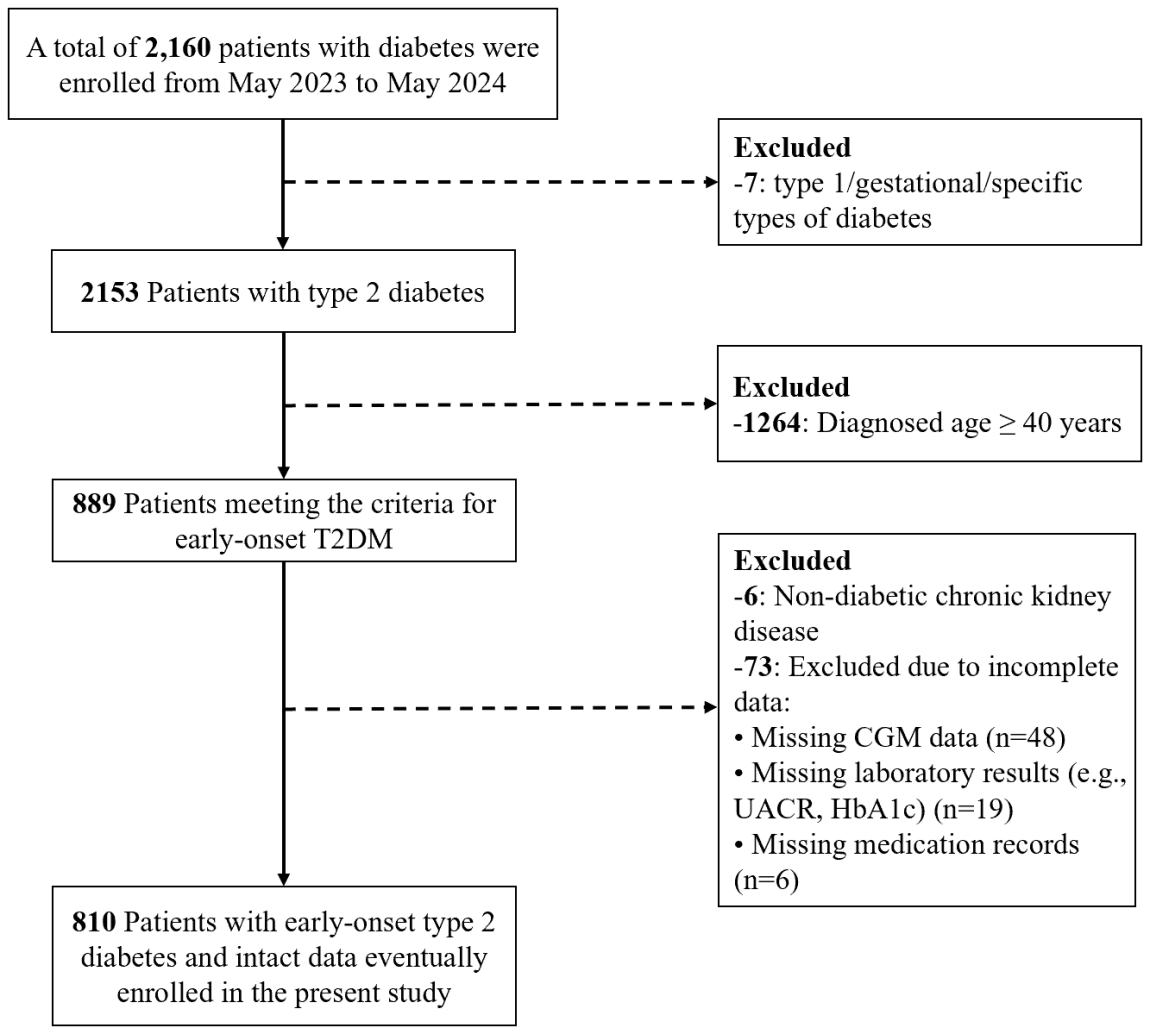
Flowchart of population enrollment. CGM, continuous glucose monitoring.

This cross-sectional study consecutively recruited patients with diabetes from the Second Hospital of Jilin University between May 2023 and May 2024. From this cohort, we identified individuals with early-onset T2DM, defined as an estimated age at diagnosis below 40 years, calculated as current age minus duration of diabetes. Diabetes duration was obtained from the electronic medical records, which contained the date of initial diagnosis documented by the attending physician at the time of first visit. For patients without clearly documented diagnosis dates, duration was confirmed by two independent physicians through structured patient interviews and cross-checked with available medical history, including previous hospitalization records and laboratory results. Patients with ambiguous or missing diagnostic records were excluded from the analysis. Key exclusion criteria were: (1) type 1 diabetes, gestational diabetes, or other specific types of diabetes; (2) chronic kidney disease attributable to non-diabetic causes (e.g., glomerulonephritis or interstitial nephritis), which was ruled out through comprehensive review of electronic medical records, including medical history, physical examination findings, urinalysis (presence of dysmorphic red blood cells or red blood cell casts suggestive of glomerulonephritis), serum complement levels, and renal ultrasound reports when available. For cases with atypical presentation (e.g., rapid eGFR decline without significant albuminuria, or active urinary sediment), nephrology consultation records were reviewed to confirm the absence of primary renal disease; (3) use of Chinese herbal medicines, proprietary Chinese medicines, or dietary supplements with known or potential effects on glucose metabolism, lipid profiles, or renal function within 3 months prior to enrollment; and (4) missing essential clinical or CGM data. A total of 810 eligible participants with early-onset T2DM were included in the final analysis. The participant selection process is detailed in **Figure 1**.

Regarding the 73 participants excluded due to missing data, the primary reasons were incomplete continuous glucose monitoring records or unavailable key laboratory measurements (see **Figure 1**). Given the relatively low proportion of missing data (73/889, 8.2%) and the lack of a clear pattern suggesting that the missingness was related to the outcome (early DKD), we employed a complete-case analysis strategy. We acknowledge that this approach assumes the data are missing completely at random (MCAR), and any violation of this assumption could introduce selection bias into our estimates.

The study was conducted in accordance with the principles of the Declaration of Helsinki and received approval from the Medical Ethics Committee of the Second Hospital of Jilin University (IRB No. 2024-226). The requirement for informed consent was waived by the ethics committee due to the retrospective nature of the study.

### 2.2 Data collection and endpoint definitions

Trained research staff, who had undergone a standardized training protocol, collected baseline data through the electronic medical record system. The collected data encompassed demographic characteristics, vital signs, laboratory results, comorbid conditions, and medication history. Information on smoking status, including the number of cigarettes smoked per day and the total duration of smoking (in years), was collected from medical records through a standardized questionnaire. Blood pressure was measured in the morning after at least 10 minutes of rest in a seated position, using a validated automated oscillometric device (Omron HEM-7130, Kyoto, Japan). Two measurements were taken 5 minutes apart, and the average of the two readings was used for analysis. All participants were required to fast for at least 8 hours prior to venous blood sampling in the morning. Fasting plasma glucose (FPG) was determined from venous blood samples collected after an overnight fast, using the hexokinase method on an automated biochemical analyzer. Lipids (TG, TC, HDL-C, LDL-C), UA, ALT, and AST were measured using a Hitachi LABOSPECT 008 automated biochemical analyzer (Hitachi, Tokyo, Japan). HbA1c was determined by high-performance liquid chromatography (HPLC) using a Bio-Rad D-100 analyzer (Bio-Rad Laboratories, Hercules, CA, USA). All assays were performed by trained laboratory technicians blinded to clinical status, with intra- and inter-assay coefficients of variation consistently below 5%. Random urine samples were collected while participants were in a resting state and free from fever, infection, or other inflammatory conditions. To confirm persistent albuminuria, two independent urine samples were obtained from each patient: the first during hospitalization, and the second approximately 4 weeks later at outpatient follow-up. Patients with transient albuminuria risk factors—including recent vigorous exercise (within 24 hours), acute hyperglycemia (random glucose > 13.9 mmol/L), uncontrolled hypertension (SBP ≥ 160 mmHg or DBP ≥ 100 mmHg), menstruation, urinary tract infection, or acute febrile illness—were resampled after resolution of these conditions. Urinary albumin and creatinine were measured by immunoturbidimetry and enzymatic methods, respectively, on a Beckman Coulter AU5800 analyzer (Beckman Coulter, Brea, CA, USA). The urinary albumin-to-creatinine ratio (UACR) was calculated for each sample. Medication history was recorded from inpatient electronic medical records, comprehensively recording the use of glucose-lowering agents (including insulin, metformin, SGLT2 inhibitors, and GLP-1 receptor agonists), antihypertensive drugs, and lipid-lowering therapies. The use of any prescribed lipid-lowering treatment was systematically documented as a binary variable (yes/no). Due to the retrospective nature of the data collection, detailed classification of lipid-lowering agents (e.g., statins, fibrates, ezetimibe, or prescription omega-3 fatty acids) was not consistently available for the entire cohort and thus was not included in the analysis. The estimated glomerular filtration rate (eGFR) was calculated using the Chronic Kidney Disease Epidemiology Collaboration (CKD-EPI) equation (18). In accordance with the American Diabetes Association standards (19) and aligned with the Kidney Disease: Improving Global Outcomes (KDIGO) clinical practice guidelines (20), early diabetic kidney disease (DKD) was defined as a UACR ≥ 30 mg/g (confirmed by at least two non-consecutive samples) with an eGFR ≥ 60 mL/min/1.73m^2^, thereby excluding patients with moderate to advanced chronic kidney disease.

### 2.3 Continuous glucose monitoring metrics

All enrolled patients underwent CGM using the Medtronic CGM system (Model: Medtronic REF MMT-7715) for a minimum of 7 days. The system consisted of a miniature glucose sensor inserted subcutaneously into the abdomen or upper arm, which automatically recorded interstitial glucose values every 5 minutes. Patients were instructed to perform at least four finger-stick blood glucose measurements per day for sensor calibration. Data with a recording time of less than 72 hours or with sensor data availability below 70% were excluded from analysis. The first 24 hours of CGM data were excluded to avoid potential artifacts from sensor insertion and stabilization. Data collection was conducted during hospitalization to ensure a standardized monitoring environment and to minimize potential confounding from daily activities, diet, and medication irregularities on glucose fluctuations. To ensure data quality and reliability, only monitoring periods with a sensor data availability greater than 70% were included in the final analysis. The following CGM-derived metrics were calculated from the qualified data:

TIR: Percentage of time with glucose levels between 3.9 and 10.0 mmol/L (21)

CV: Defined as the ratio of the standard deviation of glucose to the mean glucose level, expressed as a percentage (12)


GRI=(3.0×% very low glucose time)+(2.4×% low glucose time)+(1.6×% very high glucose time)+(0.8×% high glucose time)

(22)

Among these metrics, TIR, CV, and GRI were selected as the primary exposure variables because they capture distinct dimensions of glycemic control—overall control, variability, and composite hyper/hypoglycemic risk. TAR was excluded due to its inverse correlation with TIR and its incorporation into GRI; MG was excluded due to its strong correlation with TIR. Including these correlated metrics would introduce multicollinearity.

### 2.4 Statistical analyses

Continuous variables are presented as mean ± standard deviation for normally distributed data or as median (interquartile range) for skewed data. Categorical variables are expressed as frequencies with percentages. Group comparisons were performed using Student’s t-test, Mann-Whitney U test, chi-square test, or Fisher’s exact test, as appropriate.

Multivariable logistic regression was used to evaluate the associations between CGM metrics and early DKD. Candidate covariates were selected based on established clinical relevance and univariate analysis (*P* < 0.10). Three models were constructed with sequential adjustment: Model 1 was adjusted for demographic factors, including gender, smoking categories, and age. Model 2 was additionally adjusted for clinical characteristics, including systolic and diastolic blood pressure, body mass index, and disease duration. Model 3 was further adjusted for medication use (GLP-1 receptor agonists, SGLT2 inhibitors, and insulin) and laboratory parameters (uric acid, triglycerides, high-density lipoprotein cholesterol, low-density lipoprotein cholesterol, estimated glomerular filtration rate, and HbA1c). Multicollinearity among the independent variables in the fully adjusted models was assessed using variance inflation factors (VIF). A VIF > 5 was considered indicative of moderate collinearity, and VIF > 10 was considered indicative of severe collinearity. Smoking exposure was quantified using pack-years, calculated as (cigarettes per day/20) × years of smoking, and categorized into four groups: never-smokers (0 pack-years), light smokers (<10 pack-years), moderate smokers (10–20 pack-years), and heavy smokers (>20 pack-years), with never-smokers serving as the reference group in all regression models. Dose-response relationships between CGM metrics and early DKD were examined using restricted cubic splines with four knots placed at the 5th, 35th, 65th, and 95th percentiles of each metric’s distribution.

To evaluate the incremental value of CGM metrics beyond conventional risk factors, we constructed a baseline model including HbA1c, systolic blood pressure, disease duration, triglycerides, smoking categories, and ACEI/ARB use. TIR, CV, and GRI were then individually added to this baseline model. Model performance was evaluated by the area under the receiver operating characteristic curve (AUC), and AUCs were compared using DeLong’s test. Additionally, we calculated the net reclassification improvement (NRI) and integrated discrimination improvement (IDI) using the PredictABEL package (version 1.2-4) in R. For these analyses, predicted risks were categorized as low (<10%), intermediate (10–50%), or high (>50%).

Mediation analysis was performed to explore whether triglycerides may partly explain the associations between CGM metrics and early DKD. Given the cross-sectional design, this analysis was exploratory and cannot establish causal mediation. Sensitivity analyses were conducted by excluding patients on lipid-lowering therapy, excluding SGLT2 inhibitor or GLP-1 receptor agonist users, and using Poisson regression with robust standard errors. All statistical analyses were performed using R software (version 4.2.0). A two-sided *P*-value < 0.05 was considered statistically significant.

## 3 Results

### 3.1 Patient characteristics

A total of 810 patients diagnosed with early-onset T2DM were included in this study. Among them, 183 (22.59%) were identified as having early DKD, while 627 (77.41%) comprised the non-DKD group. The baseline characteristics of the participants are summarized in **Table 1** (medication use and comorbidities are presented in **Supplementary Table 1**). Compared with the non-DKD group, patients with early DKD showed significantly higher systolic blood pressure (SBP), diastolic blood pressure (DBP), TC, TG, UA, HbA1c, and FPG (all *P* < 0.05). Regarding smoking status, a significant association was observed between smoking intensity and early DKD (*P* = 0.007), with a progressively lower proportion of never-smokers and higher proportions of light, moderate, and heavy smokers in the early DKD group. Multiple CGM metrics indicated significantly poorer glycemic control in the DKD group. Specifically, TIR was significantly lower, while time above range (TAR), mean glucose (MG), standard deviation (SD), mean amplitude of glycemic excursions (MAGE), glucose management indicator (GMI), and GRI were all significantly elevated (all *P* < 0.05). In addition, CV also differed significantly between the two groups (*P* = 0.009). The proportion of patients with any measurable hypoglycemia (TBR > 0%) was significantly lower in the early DKD group than in the non-DKD group (4.37% vs. 13.24%, *P* < 0.001). Furthermore, patients with early DKD had significantly higher UACR levels and greater use of insulin and ACEI/ARB (all *P* < 0.05). No significant differences were observed in other demographic characteristics, laboratory parameters, or medication use between the two groups (all *P* > 0.05).

**Table 1:** Baseline characteristics of participants by early diabetic kidney disease status.

Characteristics	Overall (n=810)	Non-DKD (n=627)	Early DKD (n=183)	*P* value
Demographics				
Current age, years	37.00 (33.00, 42.75)	37.00 (33.00, 42.00)	38.00 (32.00, 43.00)	0.959
Age at diagnosis, years	32.50 (28.00, 36.00)	33.00 (28.00, 36.00)	32.00 (28.50, 36.00)	0.385
Female, n (%)	247 (30.49)	199 (31.74)	48 (26.23)	0.154
Anthropometrics and vital signs				
SBP, mmHg	130.00 (120.00, 133.00)	130.00 (120.00, 130.00)	130.00 (120.00, 140.00)	0.003
DBP, mmHg	80.00 (80.00, 88.00)	80.00 (80.00, 85.00)	80.00 (80.00, 90.00)	<0.001
BMI, kg/m²	27.37 (24.52, 30.04)	27.13 (24.48, 29.80)	27.70 (25.04, 30.42)	0.134
Disease duration, years	4.00 (1.00, 10.00)	4.00 (1.00, 10.00)	4.00 (1.00, 10.00)	0.675
Smoking and lifestyle				
Smoking status, n (%)				0.007
Never-smokers	565 (69.75)	450 (71.77)	115 (62.84)	
Light smokers (<10 pack-years)	137 (16.91)	104 (16.59)	33 (18.03)	
Moderate smokers (10–20 pack-years)	72 (8.89)	53 (8.45)	19 (10.38)	
Heavy smokers (>20 pack-years)	36 (4.44)	20 (3.19)	16 (8.74)	
Drinking, n (%)	205 (25.31)	155 (24.72)	50 (27.32)	0.476
Family history of diabetes, n (%)	378 (46.67)	293 (46.73)	85 (46.45)	0.946
Laboratory parameters				
TC, mmol/L	5.09 (4.32, 5.93)	5.01 (4.28, 5.80)	5.41 (4.42, 6.27)	0.002
TG, mmol/L	2.62 (1.67, 4.54)	2.48 (1.62, 4.08)	3.47 (1.85, 6.29)	<0.001
HDL-C, mmol/L	1.04 (0.89, 1.27)	1.06 (0.89, 1.27)	1.00 (0.86, 1.23)	0.078
LDL-C, mmol/L	2.96 (2.40, 3.55)	2.97 (2.41, 3.52)	2.95 (2.39, 3.62)	0.681
eGFR, ml/min/1.73m²	116.40 (105.50, 123.30)	115.40 (105.15, 123.00)	118.00 (106.20, 124.05)	0.338
UACR, mg/g	18.48 (10.21, 77.09)	11.70 (7.74, 15.93)	112.64 (50.07, 406.47)	<0.001
UA, µmol/L	357.00 (292.00, 433.00)	347.00 (290.00, 422.75)	374.00 (306.00, 449.00)	0.010
HbA1c, %	9.00 (7.40, 10.40)	8.60 (7.30, 10.20)	9.70 (8.40, 11.10)	<0.001
FPG, mmol/L	10.43 (8.03, 13.40)	10.00 (7.90, 12.88)	12.51 (9.09, 14.96)	<0.001
CGM metrics				
TIR, %	74.00 (52.00, 88.00)	77.00 (55.00, 90.00)	62.00 (44.00, 81.00)	<0.001
TAR, %	25.00 (11.25, 48.00)	22.00 (9.00, 45.00)	38.00 (19.00, 56.00)	<0.001
TBR, %	0.00 (0.00, 0.00)	0.00 (0.00, 0.00)	0.00 (0.00, 0.00)	<0.001†
TBR > 0%, n (%)	91 (11.23)	83 (13.24)	8 (4.37)	<0.001‡
CV, %	21.40 (18.12, 25.00)	21.70 (18.50, 25.15)	20.30 (17.10, 24.05)	0.009
MG, mmol/L	8.80 (7.60, 10.20)	8.50 (7.50, 10.00)	9.50 (8.50, 10.90)	<0.001
SD, mmol/L	2.20 (1.80, 2.70)	2.20 (1.75, 2.70)	2.40 (2.00, 2.90)	<0.001
MAGE, mmol/L	4.20 (3.40, 5.10)	4.10 (3.40, 5.10)	4.40 (3.60, 5.30)	0.013
GMI, %	7.10 (6.58, 7.70)	6.97 (6.54, 7.62)	7.40 (6.97, 8.00)	<0.001
GRI, %	24.00 (11.20, 48.00)	21.60 (9.20, 43.50)	34.40 (16.80, 59.20)	<0.001

Data are presented as median (IQR) or n (%). †*P* value derived from Mann-Whitney U test. ‡*P* value based on Fisher’s exact test.

SBP, systolic blood pressure; DBP, diastolic blood pressure; BMI, body mass index; TC, total cholesterol; TG, triglycerides; HDL-C, high-density lipoprotein cholesterol; LDL-C, low-density lipoprotein cholesterol; eGFR, estimated glomerular filtration rate; UACR, urinary albumin-to-creatinine ratio; UA, uric acid; HbA1c, glycated hemoglobin; FPG, fasting plasma glucose; TIR, time in range; TAR, time above range; TBR, time below range; CV, coefficient of variation; MG, mean glucose; SD, standard deviation; MAGE, mean amplitude of glycemic excursions; GMI, glucose management indicator; GRI, glycemic risk index.

### 3.2 Associations between continuous glucose monitoring metrics and albuminuria

**Table 2** summarizes the associations between CGM metrics and the UACR. Spearman correlation analysis revealed that UACR was significantly correlated with five CGM metrics: TIR (coefficient = -0.267, *P* < 0.001), TAR (coefficient = 0.268, *P* < 0.001), GRI (coefficient = 0.252, *P* < 0.001), MG (coefficient = 0.288, *P* < 0.001), and TBR (coefficient = -0.212, *P* < 0.001). After adjusting for age, sex, body mass index (BMI), systolic blood pressure (SBP), and insulin use, partial correlation analysis showed that TIR, TAR, GRI, and MG remained significantly associated with UACR: TIR (coefficient = -0.215, *P* < 0.001), TAR (coefficient = 0.221, *P* < 0.001), GRI (coefficient = 0.198, *P* < 0.001), and MG (coefficient = 0.237, *P* < 0.001). In contrast, the partial correlation between UACR and the CV was weak and not statistically significant (coefficient = -0.055, *P* = 0.195).

**Table 2:** Associations between continuous glucose monitoring metrics and albuminuria.

CGM metric	Spearman’s correlation		Partial correlation^†^	
	Coefficient	*P* Value	Coefficient	*P* Value
TIR (%)	-0.267	< 0.001	-0.215	< 0.001
TAR (%)	0.268	< 0.001	0.221	< 0.001
GRI	0.252	< 0.001	0.198	< 0.001
CV (%)	-0.079	0.061	-0.055	0.195
MG (mmol/L)	0.288	< 0.001	0.237	< 0.001
TBR (%)	-0.212	< 0.001	-0.180	< 0.001

CGM, continuous glucose monitoring; TIR, time in range; TAR, time above range; GRI, glycemic risk index; CV, coefficient of variation; MG, mean glucose; TBR, time below range.

^†^ Partial correlation analysis was adjusted for age, sex, body mass index, systolic blood pressure, and insulin use.

Given the unexpected inverse direction of the TBR-UACR association, we performed a supplementary analysis (**Supplementary Table 2**) to assess whether it was confounded by overall glycemic levels. After further adjusting for mean glucose (MG) in addition to the covariates above (age, sex, BMI, SBP, and insulin use), the inverse correlation was substantially attenuated and was no longer statistically significant (partial ρ = -0.024, *P* = 0.501). The partial correlation for TBR adjusted only for the primary covariates (without MG) is presented in **Table 2** for comparison.

### 3.3 The relationship between continuous glucose monitoring metrics and early diabetic kidney disease in early-onset type 2 diabetes

#### 3.3.1 TIR and early DKD

**Figure 2:**
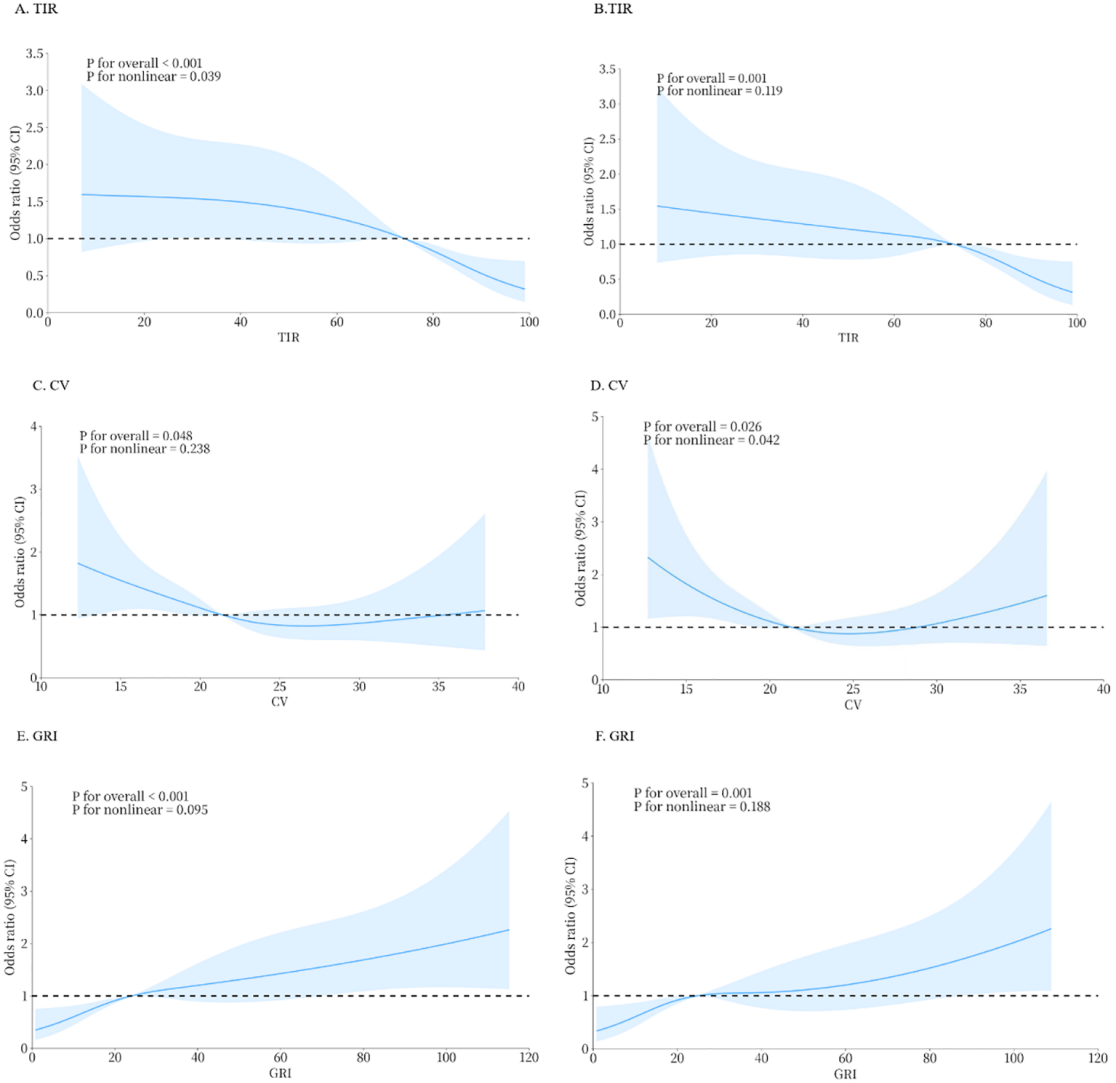
Dose-response relationships between continuous glucose monitoring metrics and the risk of early diabetic kidney disease in patients with early-onset type 2 diabetes. Restricted cubic splines were used to model potential nonlinear associations, with four knots placed at the 5th, 35th, 65th, and 95th percentiles of each metric’s distribution. The solid line represents the estimated odds ratio (OR), and the shaded band indicates the 95% confidence interval. The reference value (OR = 1) is set at the median level of each metric. The P value for nonlinearity (P-nonlinear) is displayed for each panel. **(A, B)** Association between time in range (TIR) and early DKD in the **(A)** unadjusted model and **(B)** multivariable-adjusted model. **(C, D)** Association between coefficient of variation (CV) and early DKD in the **(C)** unadjusted and **(D)** adjusted models. **(E, F)** Association between glycemic risk index (GRI) and early DKD in the **(E)** unadjusted and **(F)** adjusted models. The multivariable-adjusted model included: sex, smoking categories (never-smokers as reference; light, moderate, and heavy smokers as separate groups), current age, systolic and diastolic blood pressure, body mass index, diabetes duration, uric acid, triglycerides, high- and low-density lipoprotein cholesterol, estimated glomerular filtration rate, glycated hemoglobin A1c, and the use of SGLT2 inhibitors, GLP-1 receptor agonists, and insulin.

**Figure 3:**
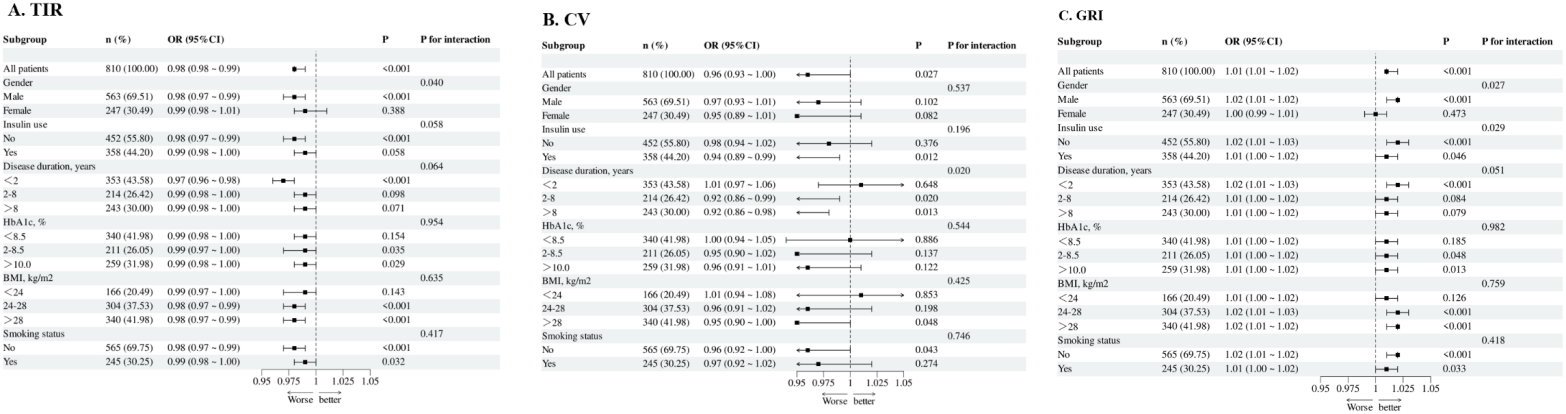
Subgroup analyses of the association between continuous glucose monitoring-related indicators and early diabetic nephropathy. **(A)** TIR, **(B)**CV, **(C)**GRI. Models were adjusted based on Model 3, with the stratification variable itself excluded from adjustment.

**Table 3** presents the results of logistic regression analyses evaluating the association between TIR and the prevalence of early DKD. TIR was identified as a strong independent protective factor against early DKD. In the fully adjusted model (Model 3), each 1% increase in TIR was associated with lower odds of early DKD (OR = 0.98, 95% CI: 0.98–0.99, *P* < 0.001). When TIR was analyzed by quartiles, participants in the highest quartile (Quartile 4) exhibited a 70% lower risk of early DKD compared to those in the lowest quartile (fully adjusted OR = 0.30, 95% CI: 0.16–0.55, *P* < 0.001). A significant inverse dose–response relationship was observed (*P* for trend < 0.001). The relationship between TIR and early DKD was further explored using restricted cubic splines (RCS). An initial nonlinear association was observed (*P* for nonlinearity < 0.05, **Figure 2A**). However, after adjustment for covariates (sex, smoking status, current age, systolic and diastolic blood pressure, BMI, diabetes duration, UA, TG, HDL-C, LDL-C, eGFR, HbA1c, and use of SGLT2 inhibitors, GLP-1 receptor agonists, and insulin), the association tended toward linearity (*P* for nonlinearity = 0.083, **Figure 2D**). Subgroup analyses showed that the protective association of TIR was generally consistent across most subgroups (**Figure 3A**). A significant interaction was observed by sex (*P* for interaction = 0.040), with a more pronounced effect in males (OR = 0.98, 95% CI: 0.97–0.99, *P* < 0.001) than in females (OR = 0.99, 95% CI: 0.98–1.01, *P* = 0.388). No other significant interactions were detected (all *P* for interaction > 0.05).

**Table 3:** Logistic regression models for the association of continuous dynamic glucose monitoring parameter with early diabetic kidney disease.

	TIR			CV			GRI		
	OR	95% CI	*P* value	OR	95% CI	*P* value	OR	95% CI	*P* value
Model 1									
per unit increase	0.98	0.98 ~ 0.99	<0.001	0.95	0.91 ~ 0.99	0.008	1.02	1.01 ~ 1.02	<0.001
Quartile 1	Reference			Reference			Reference		
Quartile 2	0.77	0.50 ~ 1.19	0.242	0.60	0.38 ~ 0.95	0.030	1.77	1.03 ~ 3.05	0.039
Quartile 3	0.54	0.35 ~ 0.85	0.008	0.65	0.42 ~ 1.02	0.059	2.31	1.36 ~ 3.92	0.002
Quartile 4	0.27	0.16 ~ 0.45	<0.001	0.51	0.32 ~ 0.81	0.005	3.49	2.10 ~ 5.80	<0.001
*P* for trend			<0.001			0.008			<0.001
Model 2									
per unit increase	0.98	0.97 ~ 0.99	<0.001	0.95	0.92 ~ 0.99	0.019	1.02	1.01 ~ 1.03	<0.001
Quartile 1	Reference			Reference			Reference		
Quartile 2	0.73	0.47~ 1.16	0.187	0.57	0.35 ~ 0.92	0.022	1.92	1.09 ~ 3.39	0.023
Quartile 3	0.50	0.31 ~ 0.81	0.004	0.61	0.39 ~ 0.98	0.041	2.71	1.56~ 4.73	<0.001
Quartile 4	0.22	0.13 ~ 0.39	<0.001	0.54	0.33~ 0.87	0.012	4.18	2.43~ 7.22	<0.001
*P* for trend			<0.001			0.018			<0.001
Model 3									
per unit increase	0.98	0.98 ~ 0.99	<0.001	0.95	0.91 ~ 0.99	0.032	1.01	1.01 ~ 1.02	<0.001
Quartile 1	Reference			Reference			Reference		
Quartile 2	0.79	0.48 ~ 1.29	0.347	0.55	0.32 ~ 0.92	0.024	2.04	1.09~ 3.79	0.025
Quartile 3	0.64	0.38 ~ 1.08	0.098	0.63	0.38 ~ 1.05	0.077	2.22	1.19 ~4.15	0.013
Quartile 4	0.30	0.16 ~ 0.55	<0.001	0.52	0.30 ~ 0.90	0.018	3.39	1.83 ~ 6.26	<0.001
*P* for trend			<0.001			0.024			<0.001

Model1: Crude; Model2: Adjust: Gender, Smoking categories (never-smokers as reference; light, moderate, and heavy smokers as separate groups), Current age, SBP, DBP, BMI, Disease duration; Model3: Adjust: Gender, Smoking categories (never-smokers as reference; light, moderate, and heavy smokers as separate groups), Current age, SBP, DBP, BMI, Disease duration, UA, TG, HDL-C, LDL-C, eGFR, HbA1c, SGLT2i use, GLP-1RA use, Insulin use. TIR, time in range; CV, coefficient of variation; GRI, glycemic risk index. VIF, variance inflation factor. Multicollinearity was assessed using VIF for all covariates in the fully adjusted models. All VIF values were < 5 (range: 1.006–1.504), indicating no substantial multicollinearity. The VIF values for TIR, CV, and GRI were 1.222, 1.022, and 1.204, respectively.

#### 3.3.2 CV and early DKD

In the univariable analysis, CV demonstrated a negative association with the risk of DKD. In the fully adjusted model (Model 3), compared with the lowest quartile (Quartile 1) of CV, the higher quartile groups exhibited a lower risk of early DKD, with the highest quartile (Quartile 4) showing a significant 48% risk reduction (fully adjusted OR = 0.52, 95% CI: 0.30–0.90, *P* = 0.018). A significant inverse trend was also observed across quartiles (*P* for trend = 0.024). To further elucidate the underlying relationship, RCS analysis was employed. While the unadjusted model suggested a linear association (**Figure 2B**), the fully adjusted model (**Figure 2E**) suggested a U-shaped relationship (*P* for nonlinearity = 0.042). This pattern suggests a dual effect of CV on DKD risk: risk may increase both when CV falls below 20% and when it exceeds 30%. However, the wide confidence intervals at the extremes of the RCS curve indicate that estimates in these regions should be interpreted with caution. Subgroup analyses for CV are presented in **Figure 3B**. A significant interaction was observed by disease duration (*P* for interaction = 0.020). No other significant interactions were detected (all *P* for interaction > 0.05). Consistent with this pattern, increasing CV above 20%—toward the 20%–30% range—may confer a relative risk reduction compared to the high-risk state of CV < 20%, which may be associated with persistent hyperglycemia and islet cell failure. In summary, this study suggests a U-shaped relationship between CV and early DKD risk in patients with early-onset type 2 diabetes, with a potential reference range of approximately 20–30% that warrants further validation. These findings suggest caution against indiscriminately pursuing excessively low CV values in clinical practice and may offer a reference for glycemic variability management in this population.

#### 3.3.3 GRI and early DKD

GRI was identified as a strong independent risk factor for early DKD. In the fully adjusted model, each 1-unit increase in GRI was associated with 1% higher odds of early DKD (OR = 1.01, 95% CI: 1.01–1.02, *P* < 0.001). When analyzed by quartiles, participants in the highest quartile (Quartile 4) exhibited a 3.39-fold higher risk of early DKD compared to those in the lowest quartile (fully adjusted OR = 3.39, 95% CI: 1.83–6.26, *P* < 0.001), with a significant positive dose–response relationship (*P* for trend < 0.001). These results indicate that GRI effectively integrates overall glycemic risk and is strongly associated with early-stage DKD. RCS analysis revealed a linear association between GRI and early DKD in both the unadjusted model (**Figure 2C**) and after comprehensive adjustment for covariates (**Figure 2F**; *P* for nonlinearity = 0.150). Subgroup analyses for GRI are presented in **Figure 3C**. The association between GRI and early DKD was consistent across most subgroups, with GRI consistently showing a risk effect (all ORs > 1). Significant interactions were observed by sex (*P* for interaction = 0.027) and insulin use (*P* for interaction = 0.029). No other significant interactions were detected (all *P* for interaction > 0.05).

#### 3.3.4 Incremental value of CGM metrics beyond conventional risk factors

We further evaluated whether adding CGM metrics improved model discrimination beyond conventional clinical factors. As shown in **Table 4**, the baseline model incorporating conventional risk factors achieved an AUC of 0.657 (95% CI: 0.608–0.705). Adding TIR significantly improved the AUC to 0.700 (95% CI: 0.655–0.744; ΔAUC = 0.043, *P* = 0.0079 by DeLong’s test). Similarly, adding GRI significantly improved the AUC to 0.702 (95% CI: 0.657–0.746; ΔAUC = 0.045, *P* = 0.0067). In contrast, adding CV did not significantly improve the AUC (0.660, 95% CI: 0.611–0.709; ΔAUC = 0.003, *P* = 0.7396).

**Table 4:** Incremental value of CGM metrics beyond conventional risk factors.

Model	AUC (95% CI)	ΔAUC	*P* for ΔAUC^*^
Baseline model^†^	0.657 (0.608–0.705)	Reference	—
Baseline + TIR	0.700 (0.655–0.744)	+0.043	0.0079
Baseline + CV	0.660 (0.611–0.709)	+0.003	0.7396
Baseline + GRI	0.702 (0.657–0.746)	+0.045	0.0067

^†^ Baseline model included HbA1c, SBP, disease duration, TG, smoking categories (never/light/moderate/heavy), and ACEI/ARB use.

*P values were calculated using DeLong’s test for paired AUC comparison.

AUC, area under the curve; CI, confidence interval; ΔAUC, change in AUC; TIR, time in range; CV, coefficient of variation; GRI, glycemic risk index.

Consistent with these findings, NRI and IDI analyses (**Table 5**) showed that adding TIR significantly improved risk reclassification (categorical NRI = 0.078, 95% CI: 0.034–0.123, *P* = 0.0006; IDI = 0.026, 95% CI: 0.012–0.040, *P* = 0.0002). Adding GRI similarly provided significant improvement (categorical NRI = 0.072, 95% CI: 0.024–0.120, *P* = 0.0032; IDI = 0.030, 95% CI: 0.014–0.045, *P* = 0.0001). However, CV did not yield consistent improvement (continuous NRI = 0.167, 95% CI: −0.004 to 0.337, *P* = 0.055; IDI = 0.006, 95% CI: 0.000–0.013, *P* = 0.043). These findings indicate that TIR and GRI, but not CV, provide significant incremental value beyond conventional risk factors.

**Table 5:** Net reclassification improvement and integrated discrimination improvement for CGM metrics.

Comparison	NRI (Categorical)	*P* value	NRI (Continuous)	*P* value	IDI	*P* value
Baseline + TIR vs. Baseline	0.078 (0.034–0.123)	0.0006	0.375 (0.206–0.545)	<0.0001	0.026 (0.012–0.040)	0.0002
Baseline + CV vs. Baseline	0.063 (0.027–0.099)	0.0006	0.167 (−0.004–0.337)	0.055	0.006 (0.000–0.013)	0.043
Baseline + GRI vs. Baseline	0.072 (0.024–0.120)	0.0032	0.343 (0.174–0.511)	<0.0001	0.030 (0.014–0.045)	0.0001

NRI, net reclassification improvement; IDI, integrated discrimination improvement; CI, confidence interval; TIR, time in range; CV, coefficient of variation; GRI, glycemic risk index. Baseline model included HbA1c, SBP, disease duration, TG, smoking categories, and ACEI/ARB use.

### 3.4 Comparative discriminatory performance of CGM metrics

**Figure 4:**
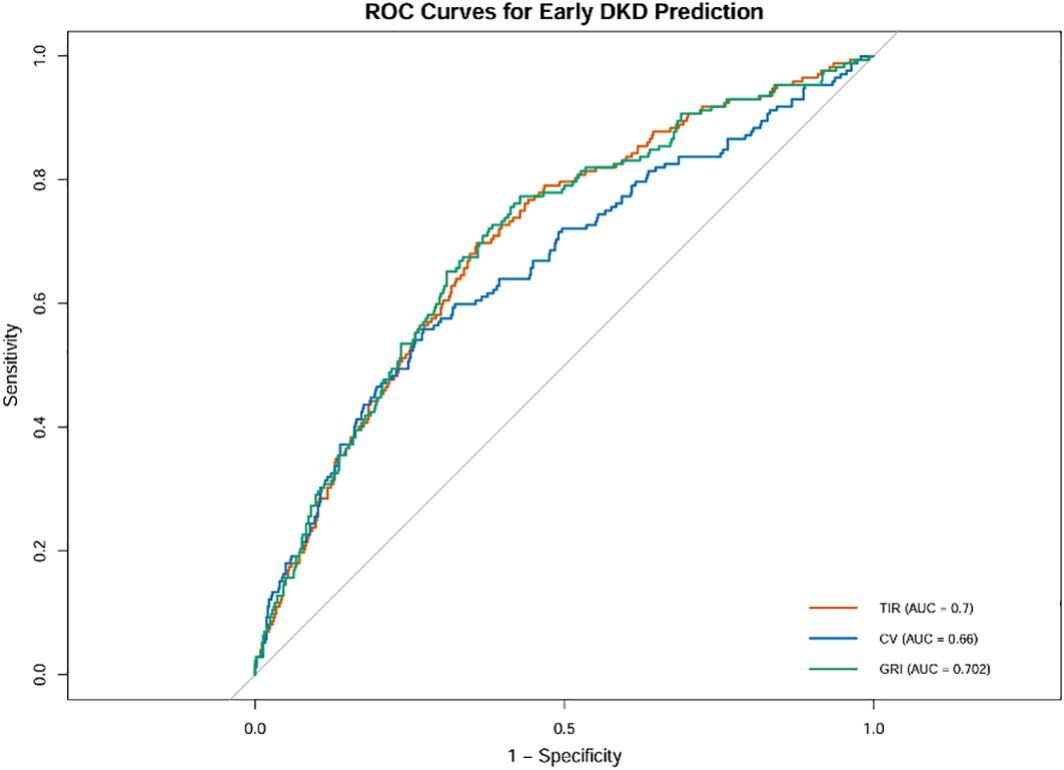
Comparison of the discriminatory performance of CGM metrics for identifying early DKD.

ROC curve analysis indicated that both TIR and GRI demonstrated moderate discriminative ability for identifying early DKD, with AUCs of 0.700 (95% CI: 0.655–0.744) and 0.702 (95% CI: 0.657–0.746), respectively. CV showed comparatively lower discriminatory performance, with an AUC of 0.660 (95% CI: 0.611–0.709). The optimal cutoff values, sensitivity, specificity, positive predictive value (PPV), and negative predictive value (NPV) for each metric are presented in **Table 6**. DeLong’s test showed no significant difference between the AUCs of TIR and GRI (*P* = 0.592), while both TIR and GRI had significantly higher AUCs than CV (*P* = 0.042 and *P* = 0.039, respectively; **Supplementary Table 3**). The modest AUC values (approximately 0.66–0.70) are consistent with the multifactorial nature of early DKD, and suggest that these CGM metrics may serve as complementary risk markers rather than standalone diagnostic tools. The comparative ROC curves of the three metrics are shown in **Figure 4**.

**Table 6:** ROC analysis of CGM metrics for identifying early DKD.

Metric	AUC (95% CI)	Optimal cutoff	Sensitivity (%)	Specificity (%)	PPV (%)	NPV (%)
TIR (%)	0.700 (0.655–0.744)	23.78	69.8	64.2	38.7	86.7
CV (%)	0.660 (0.611–0.709)	25.78	55.8	72.8	40.0	83.5
GRI	0.702 (0.657–0.746)	0.209	77.3	57.2	36.9	88.6

Optimal cutoff values were determined using the Youden index (J = sensitivity + specificity − 1). PPV, positive predictive value; NPV, negative predictive value.

### 3.5 Mediation analysis of TG in the CGM metrics-early DKD relationship

**Figure 5:**
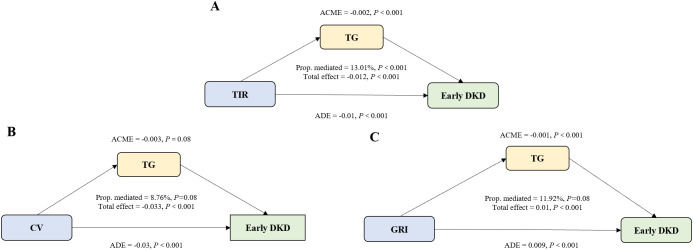
Mediation analysis of TG in the association between continuous glucose monitoring-related indicators and early diabetic nephropathy in patients with early-onset type 2 diabetes mellitus.

Bayesian mediation analysis (**Figure 5**) suggested that TG may partly explain the associations between key glycemic metrics and early DKD risk. For TIR, a significant total association was observed (estimate = -0.012, *P* < 0.001). This association was decomposed into a direct effect (ADE = -0.010, *P* < 0.001) and an indirect effect through TG (ACME = -0.002, *P* < 0.001), with TG statistically accounting for 13.01% of the total association. Similarly, a significant indirect pathway was identified for the GRI. The total association of GRI with DKD risk was significant (estimate = 0.010, *P* < 0.001), with a direct effect (ADE = 0.009, *P* < 0.001) and an indirect effect through TG (ACME = 0.001, *P* < 0.001), representing 11.92% of the total association. In contrast, although the total association of CV with early DKD risk was statistically significant, no notable indirect effect through TG was detected. This suggests that CV may influence renal risk through other pathophysiological pathways.

### 3.6 Sensitivity analyses

Several sensitivity analyses were conducted to assess the robustness of the primary findings. Excluding HbA1c from the fully adjusted model did not materially change the associations of TIR, CV, or GRI with early DKD (**Supplementary Table 4**). Similarly, the associations remained consistent after excluding users of SGLT2 inhibitors or GLP−1 receptor agonists (**Supplementary Table 5**), and Poisson regression with robust standard errors yielded results congruent with the primary logistic regression analyses (**Supplementary Table 6**). Further adjustment for lipid−lowering therapy and stratification by lipid−lowering drug use confirmed that the associations of TIR and GRI remained robust, while the association for CV was attenuated (**Supplementary Tables 7**, **S8**). In addition, excluding ACEI/ARB users did not materially change the associations for TIR or GRI (TIR: OR = 0.98, 95% CI: 0.97–0.99, *P* < 0.001; GRI: OR = 1.02, 95% CI: 1.01–1.03, *P* < 0.001). The association for CV remained in the same direction but was no longer statistically significant (OR = 0.99, 95% CI: 0.95–1.04, *P* = 0.712), likely due to reduced sample size (n=588) (**Supplementary Table 9**). In mediation sensitivity analyses restricted to participants not using lipid−lowering drugs (n=301), the indirect effects through TG remained consistent with the primary findings for TIR (proportion statistically accounted for: 9.55%, *P* < 0.001) and GRI (7.45%, *P* = 0.040), whereas no notable indirect effect was observed for CV (*P* = 0.960) (**Supplementary Table 10**). Collectively, these sensitivity analyses support the robustness of the primary findings.

## 4 Discussion

While prior studies have established links between CGM metrics and DKD in general or later-onset T2DM populations (15–17, 23), our study provides the first systematic evaluation specifically within a large, well-characterized Chinese cohort of early-onset T2DM, a population with distinct pathogenesis and high complication risk. We observed strong, independent associations of TIR and GRI with early DKD, identified a U-shaped relationship between CV and DKD risk suggesting a potential reference range of 20–30% that warrants further validation, and found that serum TG statistically accounted for a clinically significant proportion of the associations of both TIR (13.01%) and GRI (11.92%) with early DKD. These findings highlight the potential utility of CGM metrics for risk stratification in early-onset T2DM.

Substantial evidence indicates that TIR is closely associated with HbA1c (23, 24) and remains unaffected by ethnicity, anemia, pregnancy, or liver disease (25). Multiple studies have identified TIR as a marker associated with diabetic complications (11, 26–28). Our study extends these observations to early-onset T2DM, demonstrating that TIR is an independent protective factor against early DKD in this population, with a robust, linear protective effect that persisted after adjustment for HbA1c, aligning with findings from most studies in later-onset T2DM and type 1 diabetes (29–31). Dose–response and RCS analyses revealed a continuous, nearly linear protective relationship, reinforcing the principle that higher TIR is associated with greater renal protection. Subgroup analyses suggested that the protective effect of TIR may be more pronounced in patients with shorter disease duration (32), consistent with the UKPDS findings that early glycemic control provides long-term protection against microvascular complications (33). This highlights the importance of early and sustained glycemic optimization in preventing renal complications in young-onset diabetes.

The GRI, a novel composite metric integrating hyperglycemia, hypoglycemia, and glycemic variability (34), demonstrated a significant positive association with DKD in our study, even after full adjustment for HbA1c, suggesting that GRI captures glycemic risk beyond average glycemia. Acute glycemic fluctuations may be a stronger determinant of oxidative stress than chronic sustained hyperglycemia (35), suggesting that GRI provides a more comprehensive quantification of glucose-related risk than unidimensional metrics, supporting its potential utility as a risk assessment tool in early-onset T2DM. This finding is consistent with emerging evidence that higher GRI is associated with an increased risk of diabetic retinopathy (36) and atherosclerosis (37) in T2DM populations, and aligns with the conclusions of Yang et al. (31) regarding the respective roles of TIR and GRI in T2DM patients.

Although TBR showed an inverse correlation with UACR (ρ = -0.212, *P* < 0.001), this counterintuitive finding is explained by the higher mean glucose levels observed in the early DKD group, as patients with higher overall glycemia naturally spend less time in the hypoglycemic range. Importantly, this association was completely attenuated after adjustment for mean glucose (partial ρ = -0.024, *P* = 0.501), suggesting that the correlation reflects the confounding effect of overall glycemia—consistent with the “treatment-confounder trade-off,” where intensive glucose-lowering therapy improves glycemia while increasing hypoglycemia risk (38, 39) —rather than a renoprotective effect of hypoglycemia. Therefore, TBR should be interpreted as a concomitant marker of intensive treatment rather than an independent causal factor for albuminuria. Similarly, ACEI/ARB use was more common in the early DKD group (37.70% vs. 24.40%, *P* < 0.001), likely reflecting treatment for hypertension or albuminuria rather than causal exposure. Sensitivity analyses excluding ACEI/ARB users yielded consistent findings for TIR and GRI, confirming that confounding by indication did not materially affect our primary results (**Supplementary Table 8**).

In contrast to the linear relationships observed with TIR and GRI, our analysis suggested a significant U-shaped association between CV and early DKD risk, with a potential nadir at approximately 20–30% CV. However, the wide confidence intervals at the extremes of the RCS curve indicate that estimates in these regions should be interpreted with caution, and the proposed range should be considered hypothesis-generating. The right side of the curve (high CV) is consistent with the classical concept of glucose variability-induced oxidative stress and endothelial dysfunction. The left side (low CV) may reflect more advanced β-cell dysfunction, where persistently elevated glucose levels with minimal fluctuation make sustained hyperglycemia the primary driver of renal injury—a hypothesis supported by the higher HbA1c, FPG, and MG levels observed in the DKD group and by findings from Xi et al. (40) linking poorer β−cell function to lower CV values. However, this interpretation is speculative and requires validation in future studies with direct measures of β−cell function.

Our mediation analysis suggested a metric-specific role for TG in the associations between CGM metrics and early DKD. TG statistically accounted for 13.01% of the association between TIR and early DKD, and 11.92% of the association between GRI and early DKD, with sensitivity analyses confirming these findings (**Supplementary Table 11**). No notable indirect effect was observed for CV. These findings align with previous evidence that TG is independently associated with DKD (41) and that multifactorial intervention including lipid control reduces cardiorenal events (42–44). The exploratory nature of this analysis should be noted, as the cross-sectional design precludes causal mediation.

Several limitations should be acknowledged. First, the cross-sectional design precludes causal inference, and prospective studies are needed to confirm temporal relationships. Second, this was a single-center study conducted in hospitalized patients, which may not fully reflect outpatient glycemic patterns, dietary intake, physical activity, or medication adherence. Additionally, the exclusion of non-diabetic kidney disease was based on medical record review rather than renal biopsy, the gold standard for diagnosing primary glomerular diseases, and the possibility of underlying primary renal disease cannot be entirely excluded in the absence of histological confirmation. Therefore, caution is needed when generalizing these findings to the broader early-onset T2DM population, and future validation in outpatient settings is warranted. Third, although we adjusted for multiple confounders and explicitly excluded patients with recent use of herbal medicines or supplements known to affect glucose or lipid metabolism, residual confounding from unmeasured lifestyle factors—such as detailed dietary patterns, physical activity, medication adherence, and specific types, dosages, or durations of herbal product use—cannot be entirely ruled out. Fourth, the U-shaped relationship between CV and early DKD is speculative and requires mechanistic validation. Fifth, the mediation analysis was exploratory and cannot establish causal mediation due to concurrent measurement of CGM metrics, triglycerides, and DKD status. Future prospective studies with sequential measurements are needed to confirm whether improving TIR, maintaining CV within the 20–30% range, or lowering triglycerides reduces DKD risk.

## 5 Conclusion

This study suggests that CGM-derived metrics—particularly TIR and GRI—are valuable markers for risk stratification of early DKD in early-onset T2DM. The identification of a potential reference range of 20%–30% for CV provides a reference for glycemic variability control. Furthermore, the association involving TG offers a plausible biological context for early intervention. As suggested by our findings, prospective studies are needed to determine whether the integrated management of glycemia, glycemic stability, and lipid metabolism may confer renal benefits in this population.

## Data Availability

The raw data supporting the conclusions of this article will be made available by the authors, without undue reservation.
