# Supplementary material for: Association of continuous glucose monitoring metrics with early diabetic kidney disease in early-onset type 2 diabetes
Source: Front Endocrinol (Lausanne). 2026 Jul 22;17:1884596. doi: 10.3389/fendo.2026.1884596 (PMC13437500; doi:10.3389/fendo.2026.1884596)
Supplement: Supplementary file 1 [file DataSheet1.pdf]

Table S1. Medication use and comorbidities

Characteristics	Overall (n=810)	Non-DKD (n=627)	Early DKD (n=183)	*P* value
Medications				
Lipid-lowering drugs, n (%)	509 (62.84)	385 (61.40)	124 (67.76)	0.117
ACEI/ARB, n (%)	222 (27.41)	153 (24.40)	69 (37.70)	<0.001
GLP-1RA, n (%)	353 (43.58)	273 (43.54)	80 (43.72)	0.966
SGLT2i, n (%)	2 (0.25)	1 (0.16)	1 (0.55)	0.401
Metformin, n (%)	10 (1.23)	9 (1.44)	1 (0.55)	0.563
DPP4i, n (%)	5 (0.62)	4 (0.64)	1 (0.55)	1.000
Insulin, n (%)	358 (44.20)	263 (41.95)	95 (51.91)	0.017
AGI, n (%)	2 (0.25)	1 (0.16)	1 (0.55)	0.401
Comorbidities				

Characteristics	Overall (n=810)	Non-DKD (n=627)	Early DKD (n=183)	*P* value
Coronary heart disease, n (%)	2 (0.25)	2 (0.32)	0 (0.00)	1.000
Non-alcoholic fatty liver disease, n (%)	553 (68.27)	429 (68.42)	124 (67.76)	0.866

Data are presented as n (%). P values were calculated using chi-square test or Fisher‘s exact test as appropriate.
Abbreviations: ACEI/ARB, angiotensin-converting enzyme inhibitor/angiotensin receptor blocker; GLP-1RA, glucagon-like peptide-1 receptor agonist; SGLT2i, sodium-glucose cotransporter-2 inhibitor; DPP4i, dipeptidyl peptidase-4 inhibitor; AGI, α-glucosidase inhibitor.

TableS2. Supplementary Analysis of the Association Between Time Below Range and Urinary Albumin-to-Creatinine Ratio

Model	Adjustment Variables	Partial Correlation Coefficient (ρ) with UACR	*P* Value
1	None (Spearman's correlation)	-0.212	<0.001
2	Age, Sex, BMI, SBP, Insulin Use	-0.180	<0.001
3	Age, Sex, BMI, SBP, Insulin Use , MG	-0.024	0.501

Abbreviations: BMI, body mass index; SBP, systolic blood pressure; MG, mean glucose; UACR, urinary albumin-to-creatinine ratio.
Note: This analysis was conducted to test the hypothesis that the observed inverse correlation between TBR and UACR was confounded by overall glycemic exposure. UACR was log-transformed to approximate a normal distribution for parametric analysis. Model 2 corresponds to the TBR partial correlation reported in Table 2 of the main text.

Table S3. Sensitivity analysis: DeLong test comparisons of AUCs between CGM metrics for identifying early DKD

Comparison	ΔAUC	95% CI	*P* value
TIR vs. GRI	0.002	−0.008 to 0.005	0.592
TIR vs. CV	0.040	0.001 to 0.078	0.042
GRI vs. CV	0.041	0.002 to 0.081	0.039

ΔAUC, difference in area under the curve; CI, confidence interval. DeLong's test was used for paired comparisons of AUCs. AUCs were calculated based on the fully adjusted models (Model 3) in the final dataset.

Table S4. Sensitivity analysis of CGM metrics with early DKD excluding HbA1c (Model 3a)

Metric	OR (95% CI)	*P* value
TIR (per 1% increase)	0.99 (0.98–0.99)	<0.001
CV (per 1% increase)	0.97 (0.94–1.01)	0.122
GRI (per 1-unit increase)	1.01 (1.01–1.02)	<0.001

Model 3a: Adjusted for gender, smoking categories, current age, SBP, DBP, BMI, disease duration, UA, TG, HDL-C, LDL-C, eGFR, GLP-1RA use, SGLT2i use, and insulin use (excluding HbA1c).

Table S5. Sensitivity analysis: association of continuous glucose monitoring metrics with early diabetic kidney disease after excluding users of SGLT2i or GLP-1 receptor agonists (n=455)

	**TIR**			**CV**			**GRI**		
	OR	95% CI	*P* value	OR	95% CI	*P* value	OR	95% CI	*P* value
Model 1	0.99	0.98 ~ 0.99	0.002	0.96	0.92 ~ 1.00	0.073	1.01	1.01 ~ 1.02	<0.001
Model 2	0.98	0.98 ~ 0.99	<0.001	0.96	0.92 ~ 1.01	0.134	1.01	1.01 ~ 1.02	<0.001
Model 3	0.99	0.98 ~ 0.99	0.021	0.95	0.90 ~ 1.01	0.088	1.01	1.01 ~ 1.02	0.008

Model1: Crude; Model2: Adjust: Gender, Smoking, Current age , SBP, DBP, BMI, Disease duration; Model3: Adjust: Gender, Smoking, Current age , SBP, DBP, BMI, Disease duration, UA, TG, HDL-C, LDL-C, eGFR, HbA1c, Insulin use. TIR, time in range; CV, coefficient of variation; GRI, glycemic risk index

Table S6. Sensitivity analysis using poisson regression of the association between continuous dynamic glucose monitoring parameters and early diabetic kidney disease

	**TIR**			**CV**			**GRI**		
	HR	95% CI	*P* value	HR	95% CI	*P* value	HR	95% CI	*P* value
Model 1	0.99	0.98 ~ 0.99	<0.001	0.98	0.95 ~ 1.00	0.050	1.010	1.01 ~ 1.02	<0.001
Model 2	0.99	0.98 ~ 0.99	<0.001	0.98	0.95 ~ 1.01	0.105	1.010	1.01 ~ 1.02	<0.001
Model 3	0.99	0.98 ~ 1.00	0.002	0.98	0.95 ~ 1.01	0.134	1.009	1.00 ~ 1.01	<0.001

Model1: Crude; Model2: Adjust: Gender, Smoking, Current age , SBP, DBP, BMI, Disease duration; Model3: Adjust: Gender, Smoking, Current age , SBP, DBP, BMI, Disease duration, UA, TG, HDL-C, LDL-C, eGFR, HbA1c, SGLT2i use, GLP-1RA use, Insulin use. TIR, time in range; CV, coefficient of variation; GRI, glycemic risk index

Table S7. A sensitivity analysis further adjusting Model 3 for the use of any lipid-lowering drug yielded consistent results

	**TIR**			**CV**			**GRI**		
	OR	95% CI	*P* value	OR	95% CI	*P* value	OR	95% CI	*P* value
Model 1									
per unit increase	0.98	0.98 ~ 0.99	<0.001	0.96	0.93 ~ 0.99	0.027	1.01	1.01 ~ 1.02	<0.001
Quartile 1	Reference			Reference			Reference		
Quartile 2	0.77	0.50 ~ 1.19	0.242	0.60	0.38 ~ 0.95	0.030	1.77	1.03 ~ 3.05	0.039
Quartile 3	0.54	0.35 ~ 0.85	0.008	0.65	0.42 ~ 1.02	0.059	2.31	1.36 ~ 3.92	0.002
Quartile 4	0.27	0.16 ~ 0.45	<0.001	0.51	0.32 ~ 0.81	0.005	3.49	2.10 ~ 5.80	<0.001
P for trend			<0.001			0.008			<0.001
Model 2									
per unit increase	0.98	0.98 ~ 0.99	<0.001	0.97	0.94 ~ 1.00	0.068	1.02	1.01 ~ 1.02	<0.001
Quartile 1	Reference			Reference			Reference		
Quartile 2	0.76	0.48 ~ 1.19	0.232	0.57	0.36 ~ 0.93	0.023	1.96	1.11 ~ 3.44	0.019
Quartile 3	0.52	0.33 ~ 0.83	0.006	0.62	0.39 ~ 0.98	0.041	2.72	1.56~ 4.73	<0.001
Quartile 4	0.23	0.13 ~ 0.39	<0.001	0.53	0.33~ 0.87	0.011	4.16	2.41~ 7.16	<0.001

	TIR			CV			GRI		
	OR	95% CI	*P* value	OR	95% CI	*P* value	OR	95% CI	*P* value
P for trend			<0.001			0.018			<0.001
Model 3									
per unit increase	0.99	0.98 ~ 0.99	<0.001	0.97	0.93 ~ 1.00	0.081	1.01	1.01 ~ 1.02	<0.001
Quartile 1	Reference			Reference			Reference		
Quartile 2	0.81	0.50 ~ 1.32	0.400	0.56	0.33 ~ 0.93	0.026	1.98	1.07~ 3.67	0.030
Quartile 3	0.63	0.38 ~ 1.07	0.087	0.62	0.37 ~ 1.03	0.064	2.15	1.15 ~3.99	0.016
Quartile 4	0.31	0.17 ~ 0.57	<0.001	0.51	0.30 ~ 0.87	0.013	3.30	1.79 ~ 6.09	<0.001
P for trend			<0.001			0.021			<0.001

Model1: Crude; Model2: Adjust: Gender, Smoking, Current age, SBP, DBP, BMI, Disease duration; Model3: Adjust: Gender, Smoking, Current age, SBP, DBP, BMI, Disease duration, UA, TG, HDL-C, LDL-C, eGFR, HbA1c, SGLT2i use, GLP-1RA use, Insulin use, Use of lipid-lowering drugs.TIR, time in range; CV, coefficient of variation; GRI, glycemic risk index.

Table S8. Sensitivity analysis: association of continuous dynamic glucose monitoring metrics with early diabetic kidney disease after excluding users of lipid-lowering drugs (n=301)

	**TIR**			**CV**			**GRI**		
	OR	95% CI	*P* value	OR	95% CI	*P* value	OR	95% CI	*P* value
Model 1	0.99	0.98 ~ 0.99	0.022	0.98	0.92 ~ 1.03	0.390	1.01	1.01 ~ 1.02	0.028
Model 2	0.98	0.97 ~ 0.99	0.005	0.98	0.93 ~ 1.04	0.553	1.02	1.01 ~ 1.03	0.005
Model 3	0.98	0.97 ~ 0.99	0.025	0.96	0.90 ~ 1.03	0.253	1.02	1.00 ~ 1.03	0.028

Model1: Crude; Model2: Adjust: Gender, Smoking, Current age , SBP, DBP, BMI, Disease duration; Model3: Adjust: Gender, Smoking, Current age , SBP, DBP, BMI, Disease duration, UA, TG, HDL-C, LDL-C, eGFR, HbA1c, SGLT2i use, GLP-1RA use, Insulin use. TIR, time in range; CV, coefficient of variation; GRI, glycemic risk index

Table S9. Sensitivity analysis excluding ACEI/ARB users

	TIR			CV			GRI		
	OR	95% CI	*P* value	OR	95% CI	*P* value	OR	95% CI	*P* value
Model 1	0.98	0.97–0.99	<0.001	0.98	0.94–1.02	0.265	1.02	1.01–1.03	<0.001
Model 2	0.98	0.97–0.98	<0.001	0.98	0.94–1.02	0.324	1.02	1.01–1.03	<0.001
Model 3	0.98	0.97–0.99	<0.001	0.99	0.95–1.04	0.712	1.02	1.01–1.03	<0.001

Model 1: Crude. Model 2: Adjusted for gender, smoking categories (never-smokers as reference; light, moderate, and heavy smokers as separate groups), current age, SBP, DBP, BMI, and disease duration. Model 3: Adjusted for gender, smoking categories, current age, SBP, DBP, BMI, disease duration, UA, TG, HDL-C, LDL-C, eGFR, HbA1c, GLP-1RA use, SGLT2i use, and insulin use (excluding ACEI/ARB users). Data are presented as OR (95% CI).

Table S10. Sensitivity analysis: Mediation Effects of Triglycerides on the Associations Between Continuous Glucose Monitoring Metrics and Early Diabetic Kidney Disease After Excluding Users of Lipid-Lowering Drugs

CGM Metric	Total Effect (*P* Value)	Direct Effect (*P* Value)	Indirect (Mediated) Effect	*P* Value	Proportion Mediated
TIR	0.02 (<0.001)	0.02 (<0.001)	0.00	<0.001	9.55%
GRI	0.02 (<0.001)	0.02 (<0.001)	0.00	0.040	7.45%
CV	0.02 (<0.001)	0.02 (<0.001)	-0.00	0.960	-0.15%

Abbreviations: CGM, continuous glucose monitoring; TIR, time in range; GRI, glycemic risk index; CV, coefficient of variation; TG, triglycerides; DKD, diabetic kidney disease.
Note: This analysis was performed in the subgroup of participants not using any lipid-lowering drugs (n = 301) to assess the robustness of the mediation pathways against potential confounding by such pharmacotherapy. The "Total Effect" is the association between the CGM metric and early DKD; the "Direct Effect" is this association after accounting for the mediator (TG); the "Indirect Effect" is the component of the total effect that operates through TG (i.e., the mediation effect).

Table S11. Mediation analysis of triglycerides in the association between continuous glucose monitoring metrics and early diabetic kidney disease (full cohort, n = 810)

CGM Metric	Total Effect	Direct Effect	Indirect (Mediated) Effect	*P* value	Proportion Mediated
TIR	−0.012	−0.010	−0.002	<0.001	13.01%
GRI	0.010	0.009	−0.001	<0.001	11.92%
CV	−0.033	−0.030	−0.003	0.08	8.76%

Note: This analysis was performed in the full cohort (n = 810). Proportions for CV are not reported as the indirect effect was not statistically significant.
